# Genomic Characterization of *Lactiplantibacillus plantarum* Strains: Potential Probiotics from Ethiopian Traditional Fermented Cottage Cheese

**DOI:** 10.3390/genes15111389

**Published:** 2024-10-29

**Authors:** Seyoum Gizachew, Ephrem Engidawork

**Affiliations:** 1 Department of Pharmacology and Clinical Pharmacy, School of Pharmacy, College of Health Sciences, Addis Ababa University, Addis Ababa P.O. Box 9086, Ethiopia; seyoumadall@gmail.com; 2 Department of Bioscience Engineering, Faculty of Sciences, University of Antwerp, 2000 Antwerp, Belgium

**Keywords:** *Lactiplantibacillus plantarum*, genome sequencing, comparative genome analysis, safety, bacteriocins, probiotic marker genes

## Abstract

Background: *Lactiplantibacillus plantarum* is a species found in a wide range of ecological niches, including vegetables and dairy products, and it may occur naturally in the human gastrointestinal tract. The precise mechanisms underlying the beneficial properties of these microbes to their host remain obscure. Although Lactic acid bacteria are generally regarded as safe, there are rare cases of the emergence of infections and antibiotic resistance by certain probiotics. Objective: An in silico whole genome sequence analysis of putative probiotic bacteria was set up to identify strains, predict desirable functional properties, and identify potentially detrimental antibiotic resistance and virulence genes. Methods: We characterized the genomes of three *L. plantarum* strains (54B, 54C, and 55A) isolated from Ethiopian traditional cottage cheese. Whole-genome sequencing was performed using Illumina MiSeq sequencing. The completeness and quality of the genome of *L. plantarum* strains were assessed through CheckM. Results: Analyses results showed that *L. plantarum* 54B and 54C are closely related but different strains. The genomes studied did not harbor resistance and virulence factors. They had five classes of carbohydrate-active enzymes with several important functions. Cyclic lactone autoinducer, terpenes, Type III polyketide synthases, ribosomally synthesized and post-translationally modified peptides-like gene clusters, sactipeptides, and all genes required for riboflavin biosynthesis were identified, evidencing their promising probiotic properties. Six bacteriocin-like structures encoding genes were found in the genome of *L. plantarum* 55A. Conclusions: The lack of resistome and virulome and their previous functional capabilities suggest the potential applicability of these strains in food industries as bio-preservatives and in the prevention and/or treatment of infectious diseases. The results also provide insights into the probiotic potential and safety of these three strains and indicate avenues for further mechanistic studies using these isolates.

## 1. Introduction

Lactic acid bacteria (LAB) are a diverse group of bacteria producing lactic acid as the main end-product of carbohydrate fermentation [1] and are ubiquitously distributed in nature [2]. They include several genera, such as the emended genus *Lactobacillus* [3], *Lactiplantibacillus*, *Lacticaseibacillus*, *Limosilactobacillus*, *Streptococcus*, *Pediococcus*, *Leuconostoc*, and *Weissella* [3,4]. They are being continuously researched due to probiotic attributes and potential health benefits. Probiotics are live microorganisms that, when administered in adequate amounts, confer a health benefit to the host [5]. Probiotic bacteria have positive impacts on the immune system and on the composition and functioning of the gut microbiota. Moreover, the biosynthesis of vitamins has been assumed to be among the causal relationships of the health benefits of probiotics [6]. LAB as probiotics are used to prevent and/or treat gastrointestinal disorders, and a wealth of evidence emerging from studies also indicates their anti-cancer activity [7,8].

*Lactiplantibacillus plantarum* [3] is a gram-positive, non-motile, non-spore-forming, microaerophilic, and mesophilic bacterium that belongs to the group LAB [9,10]. It is one of the most adaptable LAB species, as evidenced by its ability to inhabit a wide range of niches such as the gastrointestinal, vaginal and urogenital tracts, meat, fish, fermented vegetables, wine, and dairy products [1,10,11]. It is widely used in industrial fermentation and as probiotics since it is “Generally Recognized as Safe” (GRAS) and has a Qualified Presumption of Safety (QPS) status [12,13,14].

Throughout the last century, documented health-promoting and functional properties of *L. plantarum* strains have generated attention for their applications [10]. Beneficial properties attributed to *L. plantarum* are diverse, varying from its use in the fermentation of dairy products such as cheese, kefir, sauerkraut, fermented meat products, fermented vegetables, and beverages to its cholesterol-lowering activity, enhancement of the intestinal barrier, immunomodulation, and prevention of bacterial and viral infections [1,15]. Its antibacterial properties are also interesting for food safety, as in the biopreservation technology [1].

The European Food Safety Authority (EFSA) requires unequivocal taxonomic identification at the strain level in whole genome sequence (WGS) analysis of microorganisms intentionally used in the food chain [16]. The WGS analysis also provides a better understanding of the relation between strains’ genotypic and phenotypic profiles and, thus, is required to better understand strain features [17]. Although LAB are GRAS, there are rare cases of the emergence of some infections and antibiotic resistance [18]. For this reason, data obtained from WGS analysis are required for the unequivocal taxonomic identification of the strains. Moreover, the analysis can provide valuable information regarding the potential functional traits, virulence factors, resistance to antimicrobials, and the production of toxic metabolites [16]. In this study, we aimed to taxonomically identify and explore the genome of the three strains (54B, 54C, and 55A) isolated in our previous study [19] using in silico WGS analysis as per the EFSA recommendations [16].

## 2. Materials and Method

### 2.1. Bacterial Strains, Growth Conditions, and Genomic DNA Extraction

Three isolates (*L. plantarum* 54B, 54C, 55A) originally isolated from Ethiopian traditional cottage cheese were selected based mainly on their performance in antimicrobial and cell culture assays. The source, Ethiopian traditional cottage cheese, was produced by heating a spontaneously fermented (18–24 h) and defatted cow milk at the household level, and the strains were characterized in our previous study [19]. Strains were revived from −80 °C glycerol stocks on de Man, Rogosa, and Sharpe (MRS) (a selective medium used to enrich LAB [20]) plates and incubated for 48 h at 37 °C. Single colonies were cultivated in MRS broth for 24 h at 37 °C. Total DNA content was extracted using a modified protocol based on Alimolaei and Golchin [21]. Briefly, 1.5 mL of overnight culture was transferred twice to two sterile Eppendorf tubes, and 1.5 µL of ampicillin (100 mg/mL) was added and incubated at 37 °C for 1 h. The culture was then spun down at 12,000× *g* for 3 min to remove the supernatant, and the pellet was washed 3× with 1 mL of NaCl-EDTA. The pellets present in both Eppendorf tubes were pooled into one Eppendorf tube. The cell pellets were resuspended in 100 µL of NaCl-EDTA and 100 µL of lysozyme (10 mg/mL), and 1 µL RNase (20 mg/mL) was added to the tube and incubated at 37 °C with periodic shaking for 1 h. Following this, 229 µL of NaCl-EDTA, 50 µL of 10% SDS, and 20 of µL Proteinase K were added, vortexed, and incubated at 55 °C for 1 h. Then, 200 µL of cold protein precipitation solution was added and vortexed at maximum speed for 20 sec. The mix was then centrifuged at 12.000× *g*, at 4 °C, for 3 min after being placed on ice for 5 min. The supernatant was transferred to a clean 1.5 mL tube, centrifuged again (12,000× *g*, 4 °C, 3 min), and the supernatant was transferred to a clean 1.5 mL tube. The DNA was precipitated with 600 µL ice-cold isopropanol and centrifuged at 12,000× *g*, at 4 °C, for 3 min to discard the supernatant. The pellet was then washed with 600 µL fresh 70% ethanol, the supernatant was discarded, and the tube was left to air-dry. Finally, the pellet was dissolved in 50 µL H_2_O, incubated at 55 °C for 5 min, and stored at −20 °C. DNA samples in the range of 25–50 ng/µL (measured with Qubit), with a minimum volume of 20 µL, were sent for WGS.

### 2.2. Genome Sequencing, Assembly and Annotation

High molecular weight genomic DNA of the isolates was then further processed for sequencing using Nextera library prep and MiSeq sequencing (Illumina) at the lab of Medical Microbiology, University of Antwerp. After sequencing, the raw reads were demultiplexed, and the barcode sequences were removed. The raw reads were also subjected to adapter cutting and quality filtering using the Fastq Utilities Service (with Trim, Paired Filter, FastQC, and Align pipeline options) of the bacterial and viral-bioinformatics resource center (bv-brc).org [22] using the default parameters. The reads were de novo assembled into contigs using SPAdes (3.12.0) [23] with default parameters in the Shovill (1.0.0) pipeline. Quality and completeness were assessed using CheckM (completeness > 94% required) [24]. Annotation was performed with Prokka (1.12) [25].

### 2.3. Bioinformatic Tools for Comparative Genomics Studies

Comparative genomic analysis was performed using bv-brc.org [22]. The bv-brc Taxonomic Classification service (accessed on 8 September 2024) was used to identify species of the strains using WGS reads following the pipeline established by Lu et al. [26] that utilizes the Kraken 2 taxonomic classification system [27]. The pipeline uses exact-match database queries of k-mers. Sequences are classified by querying the database for each k-mer in a sequence and then using the resulting set of lowest common ancestor (LCA) taxa to determine an appropriate label for the sequence. A comparative phylogenetic tree was constructed in bv-brc using the “Bacterial Genome Tree” tool (accessed on 24 August 2024), which generates a phylogenetic tree using the codon tree method for the three novel *L. plantarum* strains (54B, 54C, and 55A) together with 23 previously published *L. plantarum* isolates from different spectrum of niches. The Codon Tree pipeline generates bacterial phylogenetic trees by using the amino acid and nucleotide sequences from a defined number of the bv-brc global Protein Families (PGFams), which are picked randomly to build an alignment and then generate a tree based on the differences within those selected sequences. The support values in the system are generated using 100 rounds of the “Rapid” bootstrapping option [28] of RAxML (8.2.11). The ‘Comparative Systems Service’ of the bv-brc (accessed on 1 May 2024) was also utilized to perform pathways analyses, to compare protein families among the genomes included in the analysis, and to mine the presence or absence of the ‘probiotic marker genes’ in the genomes studied. The Pathway Comparison Tool is based on the Rapid Annotation using Subsystem Technology tool kit (RASTtk) [29] annotations. It allows the identification of the presence or absence of metabolic pathways based on taxonomy, pathway ID, EC number, pathway name, and/or specific annotation type. The Average Nucleotide Identity (ANI) was calculated by using FASTANI v1.33. The ‘Variation Analysis’ service of the bv-brc was used to measure genetic variations of single nucleotide polymorphisms (SNPs) between the isolates *L*. *plantarum* 54B and 54C.

### 2.4. Prediction of Putative Biosynthetic Gene Clusters of Bioactive Compounds

To predict genes coding for different types of biosynthetic pathways involved in the production of secondary metabolites (SMs), antiSMASH 7.0 (Antibiotics and Secondary Metabolite Analysis Shell) was utilized (accessed on 10 August 2023) [30]. More in-depth analyses were performed in antiSMASH for biosynthetic gene clusters (BGCs) encoding non-ribosomal peptide synthetases (NRPSs), polyketide synthases (PKSs), ribosomally synthesized and post-translationally modified peptides (RiPPs), and RiPP-like molecules. The annotated genome FASTA file of the isolates was used as the input file, and default antiSMASH features were assumed during the analysis. Riboflavin metabolism pathway encoding genes were predicted by utilizing the bv-brc’s ‘Comparative Systems’ service. A bacteriocin gene detection software, BAGEL Version 5, was also used for the generation of data on bacteriocin-encoding genes [31].

### 2.5. Carbohydrate–Active Enzyme Analysis

CAZymes of the isolates were searched against the CAZy database (http://www.cazy.org/, accessed on 20 October 2021). The database mainly included glycoside hydrolases (GHs), glycosyltransferases (GTs), carbohydrate esterases (CEs), carbohydrate-binding enzymes (CBMs), auxiliary activity (AAs), and polysaccharide lyases (PLs) [11].

### 2.6. Virulome and Resistome Predictions

The genomes were assessed for safety using several tools recommended in the EFSA guidance document [32]. ABRIcate v1.0.0 (https://github.com/tseemann/abricate; accessed on 20 October 2021) and ResFinder (https://cge.cbs.dtu.dk/services/ResFinder-4.0/; accessed on 20 October 2021) [33] were employed to identify the resistome in the genomes of the isolates with their default parameters. ABRIcate and VFDB v5 (virulence factor database, http://www.mgc.ac.cn/VFs/main.htm, accessed on 20 October 2021) were also employed to predict putative virulence factors with their default parameters [16,34].

### 2.7. Genome Sequences Data Accession Number

The raw sequences *for L. plantarum* strains (54B, 54C, and 55A) were submitted to the Sequence Read Archive of the National Center for Biotechnology Information with accession number PRJNA1175724 and can be accessed at https://www.ncbi.nlm.nih.gov/sra/PRJNA1175724, accessed on 20 October 2021. The sample accession numbers are as follows: SAMN44370248 for *L. plantarum* 54B, SAMN44370249 for *L. plantarum* 54C, and SAMN44370250 for *L. plantarum* 55A.

## 3. Results

### 3.1. General Genomic Characteristics of the Strains

The chromosomal properties, quality control statistics, and identification to the species level of the three *L. plantarum* isolates (54B, 54C, and 55A) sequenced in this study are summarized in Table 1. Assembly of the raw reads generated bacterial chromosomes, each with a size similar to that previously reported for sequenced *L. plantarum* isolates (range of 3–3.6 Mbp) [35]. The two isolates (54B and 54C) possessed a genome length of 3.39 and 3.37 Mbp, respectively, while the isolate 55A possessed a genome length of 3.29 Mbp. The two isolates (54B and 54C) also possessed approximately the same coding sequence (CDS) (3259 and 3230, respectively) and the same GC content (44.3%), although isolate 55A contained lower CDS (3108) and relatively higher GC content (44.5%). However, the number of tRNA, rRNA, and tmRNA genes was found to be the same among the isolates (Table 1). The bv-brc taxonomic classification service that used the WGS reads to identify species of the strains showed that all three isolates belong to the *L. plantarum* species. Here, the completeness percentage was found to be 99.07% for all the genomes sequenced.

### 3.2. Comparative Genomic Analysis

**Figure 1:**
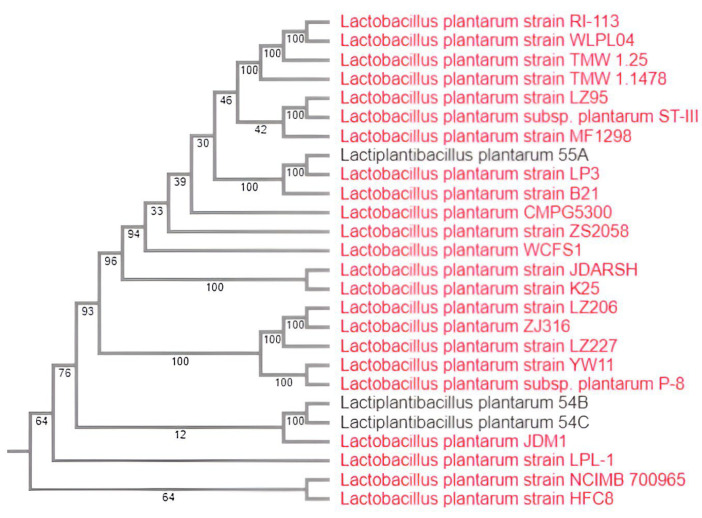
Phylogenetic analysis of *L. plantarum* 54B, 54C, and 55A with 23 other *L. plantarum* genomes. This phylogenetic tree was generated in the BV-BRC using the “Bacterial Genome Tree” tool for the 23 representative *L. plantarum* genomes (6 human isolates, 6 dairy isolates, 4 meat products isolates, and 7 isolates from different sources) and the 3 genomes under study. Parameters: Max allowed deletions = 0; Max allowed duplications = 0; Single-copy genes found = 100; Number of protein alignments = 100; Alignment program = mafft; Number of aligned amino acids = 39,007; Number of CDS alignments = 100; Number of aligned nucleotides = 117,021; Best protein model found by RAxML = DUMMY2; Branch support method = RAxML Fast Bootstrapping; RAxML likelihood = −329,039.7462.

The genomic diversity of the three novel *L. plantarum* strains, together with 23 previously published *L. plantarum* isolates from different spectra of niches, was investigated by constructing comparative phylogenetic trees to reveal the evolutionary relationship between the three *L. plantarum* strains (54B, 54C, and 55A) with other *L. plantarum* strains (Supplementary Table S1). The phylogenetic tree revealed that the strains 54B and 54C showed a high degree of similarity with each other (Figure 1). The strain 55A formed a different cluster than the 54B and 54C and displayed a high degree of similarity with the strain *L. plantarum* LP3, which was originally isolated from vegetables [36]. Comparative analysis of these 26 *L. plantarum* genomes (23 previously published and the 3 from the present study) was performed using the ‘Comparative systems service’ of bv-brc.org. The analysis resulted in a total of 5137 protein families present across 26 strains, of which 1780 (34.65%) constitute the core genome and are present in at least one copy in all examined strains, whereas 3357 (65.35%) constituted families containing accessory gene functions only present in some strains. The comparative genomic analysis was also performed among the three genomes and the model probiotic *L*. *plantarum* WCFS1 to compare protein families among the four genomes. The analysis returned a total of 3080 protein families present across the four strains, of which 2013 (65.35%) constitute the core genome and are present in at least one copy across the examined strains, whereas 1067 (34.65%) constituted families containing accessory gene functions only present in some strains, indicating the three genomes share more genes with the model probiotic. The ANI value for our three isolates among themselves and between them and *L. plantarum* WCFS1 (Table 2) was also calculated. The ANI value for the *L. plantarum* 54B vs. 54C was found to be 99.9941%, indicating that they are the most related strains. The analysis of genetic variations of SNPs between closely related isolates *L. plantarum* 54B and 54C indicated that there are 111 single nucleotide variations (SNVs) between the genomes.

### 3.3. Analysis of ‘Probiotic Marker Genes’

Studies have suggested a set of genes associated with resistance to stress, active metabolism in the host, adhesion, and immunomodulation as genes with probiotic properties [37,38,39]. Based on such findings, Carpi et al. [40] generated an updated list of ‘probiotic marker genes’, including genes putatively accountable for stress resistance (osmotic, acid, oxidative, temperature), adhesion capacity, intestinal persistence and bile salt hydrolase activity. In total, 75 probiotic marker genes have been reported, of which about 70% correspond to genes located in the core/soft core genome [40], while 12 genes (Supplementary Table S2) are located in the shell/cloud genome.

**Figure 2:**
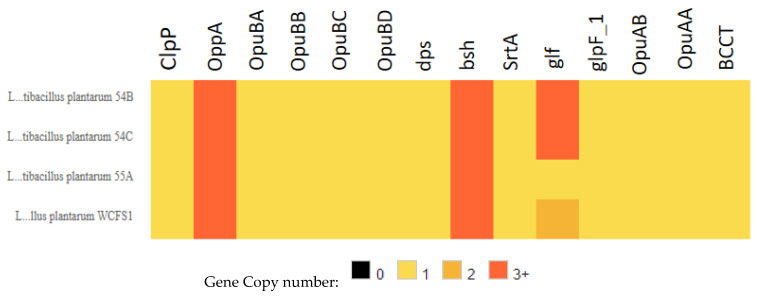
Analysis of the presence of probiotic marker genes from the cloud genome proposed by Carpi et al. [40] for the species *L. plantarum*. Numeric values refer to the number of gene copies.

Here, we analyzed the presence or absence of these probiotic marker genes in the genomes of the four strains evaluated in this work (*L. plantarum* 54B, 54C, 55A, and WCFS1). As expected, most of the core genome ‘probiotic marker genes’ were found in *L. plantarum* 54B, 54C, 55A, and WCFS1 (Supplementary Table S3). The analysis also revealed the presence of the probiotic marker genes of the cloud genome, including genes for osmotic stress, bile and acid resistance, and gut persistence in the genomes studied (Figure 2). The genes *bshA* (bile salt hydrolase), *gbuB* (Glycine betaine/carnitine transport permease protein GbuB), *gla2* (Glycerol facilitator-aquaporin gla), and *xylA* (xylose isomerase) were not detected in any of the four genomes.

### 3.4. Prediction of Secondary Metabolites and Bioactive Products

**Figure 3:**
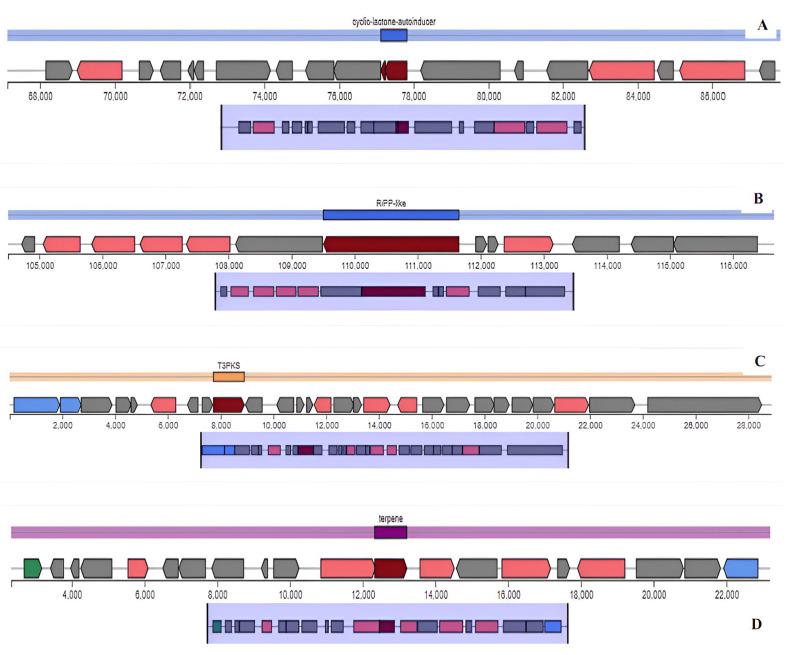
The antiSMASH system predicted bacteriocins and secondary metabolite-producing regions in the genome of *L. plantarum* 55A: Red (core biosynthetic genes), pink (additional biosynthetic genes), blue (transport-related genes), green (regulatory genes), grey (other genes), and black (resistance). (**A**) Cyclic lactone autoinducer; (**B**) RiPP-like; (**C**) Type III PKS; (**D**) Terpene.

**Figure 4:**
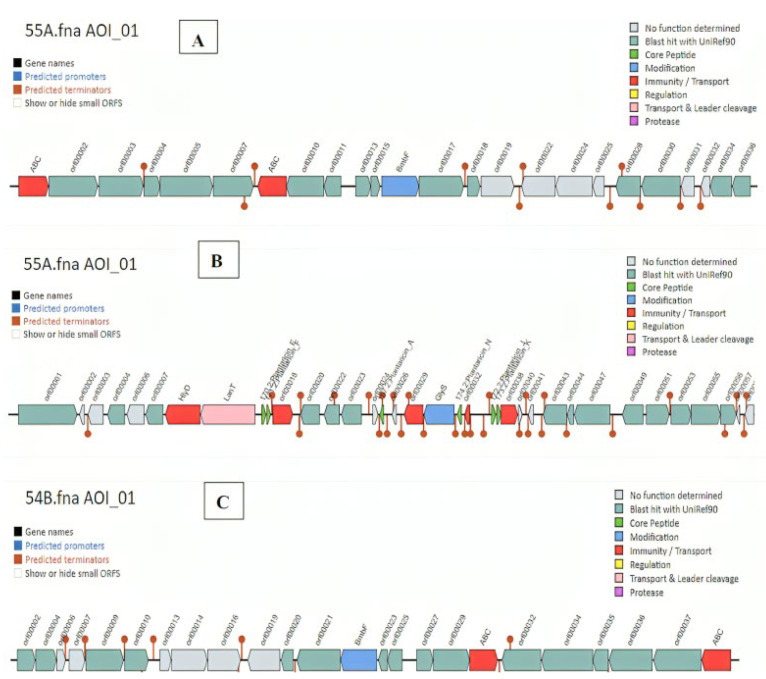
Identification of chromosomal gene clusters of the *L. plantarum* 55A containing genes encoding sactipeptides (**A**), plantaricin-like proteins (**B**), and chromosomal gene clusters of the *L. plantarum* 54B containing genes encoding sactipeptides (**C**) using BAGEL v5 software.

Bacteriocins represent a significant class of antimicrobial peptides synthesized by LAB. In this study, the antiSMASH system predicted four fundamental areas that produce bacteriocins and secondary metabolites in the genome of *L. plantarum* 55A; region 1.1 (RiPP: cyclic-lactone autoinducer; location: 67,119–87,824 nt; total: 20,706 nt); region 2.1 (RiPP: RiPP-like; location: 104,505–116,655 nt; total: 12,151 nt); region 17.1 (PKS: T3PKS; location: 1–28,896 nt; total: 28,896 nt) and region 25.1 (terpene: terpene; location: 2330–23,211 nt; total: 20,882 nt) (Figure 3). An application of the bacteriocin gene detection software BAGEL5 also revealed the presence of two areas of interest for sactipeptides and plantaricins in the genome of *L. plantarum* 55A. The genes encoding these sactipeptides are located at positions from 18,188 bp to 38,188 bp in the genome of *L. plantarum* 55A (Figure 4). The BAGEL5 analysis of the *L. plantarum* 55A also demonstrated the presence of several genes encoding bacteriocin-related proteins associated with the production and immunity to these compounds (Figure 4). There are six bacteriocin-like structures encoding genes, *PlnA*, *PlnE*, *PlnF*, *PlnJ*, *PlnK*, and *PlnN,* in the genome of *L. plantarum* 55A. These genes are located at positions from 101,924 bp to 131,362 bp.

The antiSMASH system also predicted three fundamental areas that produce bacteriocins and secondary metabolites in the genome of *L. plantarum* 54B (Table 3. The genome of *L. plantarum* 54C was also observed to have exactly the same bacteriocins and secondary metabolites profile as *L. plantarum* 54B; the minor differences included the location of the PKS region and its total nucleotide (Table 3). The BAGEL5 analysis also showed the presence of an identical area of interest for sactipeptides in the genomes of *L. plantarum* 54B and 54C, with its location from 186,914 bp to 206,914 bp (Figure 4).

Genome analysis with the KEGG (Kyoto Encyclopedia of Genes and Genomes) database revealed that all of our *L. plantarum* genomes harbor all genes required for riboflavin production, which was confirmed in the gene list annotated in the comparative systems service pathway analysis tool (Table 4). The genome of *L. plantarum* 54C was shown to have the same genes involved in riboflavin production as *L. plantarum* 54B; the only difference is the location of Alkaline phosphodiesterase I (EC 3.1.4.1)/Nucleotide pyrophosphatase (EC 3.6.1.9) gene in which it starts on bp 34,650 for 54B and on 95,503 bp for 54C (Table 4). The analysis also revealed the presence of identical enzymes of riboflavin production in the genome of *L. plantarum* 55A with those of *L*. *plantarum* 54B and 54C, the differences being the position of genes and duplication of 3,4-dihydroxy-2-butanone 4-phosphate synthase (EC 4.1.99.12)/GTP cyclohydrolase II (EC 3.5.4.25) gene in the genome of *L. plantarum* 55A (Table 4). Genome analysis with the KEGG database also showed that strains *L*. *plantarum* 54C and 55A have all enzymes of the Folate biosynthesis pathway. Whereas, strain *L*. *plantarum* 54B lacks the enzyme E.C. 2.7.6.3 (2-amino-4-hydroxy-6-hydroxymethyl dihydropteridine diphosphokinase).

Here, we report that the bv-brc.org analyses revealed the three strains and the model probiotic *L. plantarum* wcfs1 harbored genes coding for the protein tyrosine kinase and phosphoprotein phosphatase, which are associated with T cell receptor signaling pathways.

### 3.5. Carbohydrate-Active Enzyme

The analysis of CAZymes revealed that the *L. plantarum* 54B and 54C genomes each contained 91 genes in the five CAZymes gene families: 36 GT, 41 GH, 2 AA, 3 CBMs, and 9 carbohydrate CE genes. At the same time, CAZymes analysis on the genome of *L. plantarum* 55A revealed that it contained 90 genes in the five CAZymes gene families: 31 GT, 47 GH, 2 AA, 2 CBM, and 8 CE genes. Previous genomic analysis found that *L. plantarum* has five different families of enzymes involved in carbohydrate metabolism: GH, GT, CE, CBM and AA enzymes [41]. We found that the most abundant CAZymes genes in the *L. plantarum* strain genomes belonged to the GH family, followed by the GT and CE families. The most abundant GT families in *L. plantarum* strains were GT2 and GT4, whereas the most abundant GH families were found to be GH1, GH13, and GH25.

### 3.6. Prediction of Antibiotic Resistance Genes and Virulence Factors

Although *L. plantarum* is a species with QPS status, with the aim of using the strains under study in food applications, their genomes were evaluated to cover all the safety concerns as recommended by EFSA Guidance for the characterization of microorganisms used as food additives, in animal feed, and as producing organisms [32]. Here, in our analysis, both ABRIcate and ResFinder revealed that the genomes of the strains under study harbored no antibiotic-resistant genes. ABRIcate and VFDB analyses also showed that the strains under study harbored no putative virulence factors. These findings suggest the strains’ potential safety for food and other applications.

## 4. Discussion

This study reports the draft genome sequence of three *L. plantarum* strains (54B, 54C, and 55A) isolated from the Ethiopian traditional cottage cheese samples and reported to have antipathogenic and immunostimulatory activities [19], with insights into the potential probiotic functions of these strains based on the presence of putative beneficial genes and absence of genes of safety concern. Notably, the food-dwelling *L. plantarum* strains studied are representative of LAB isolates that are naturally consumed at very high levels (~10^8^–10^9^ per gram) in cottage cheese [19], and it is, therefore, crucial to understand the genetic makeup of these strains and their potential effect on the host. Via this in silico genomic analysis, this study aimed to obtain insights into the key genes and predict the functionality and safety concerns of these strains to foster future studies and applications.

Assembly of the raw reads led to the generation of bacterial chromosomes, each with a size similar to that reported before for sequenced *L. plantarum* isolates (range: 3–3.6 Mbp), which is high compared to the results for other LAB [35]. With the completeness of 99.07% for all the genomes sequenced and the sequences produced 101–104% of the median total length of *L. plantarum* genomes, we report a genome sequence of comparable size with other genomes of the bacterium. The phylogenetic analysis assessed the genetic relatedness between the three strains and 23 complete sequences of *L. plantarum*. The relationship between strain origin and gene content was assessed by analyzing the protein families. The result of this analysis indicates two major findings: first, there is significant diversity among the genomes of *L. plantarum* strains since 65.35% of the protein families assessed corresponded with the variable genome, and, second, no origin of isolation-dependent grouping was recorded among the strains. Particularly, one of the three strains examined was positioned in a different clade, although it was isolated from the same source. These findings are in line with another study [10] that evaluated 42 strains isolated from different sources to study the link between intra-species genetic variability and their environmental origin.

Using 127 full genomes from open-access archives, a recent study performed a thorough pan-genomic analysis of *L. plantarum* [40]. Based on previous works suggesting that genes linked to stress tolerance, active metabolism in the host, adhesion, and intestinal persistence are involved in the beneficial properties of lactobacilli, this study identified 75 “probiotic marker” genes in the genomes of *L. plantarum*. Of the 75 “probiotic marker” genes, 70% corresponded to genes located in the core and soft-core genome. Most of these genes were found in all the *L. plantarum* genomes used in this study [40]. On the other hand, 12 genes (bshA, *ClpP1*, *oppA3*, *oppA4*, *xylA*, *srtA*, *gbuB*, *gla2*, *dps*, *glf*, *glpF1,* and *cbh/bsh*) were located in the shell and cloud genome, showing that they were only present in some strains [40]. The genes *gbuB*, *bshA*, *gla2,* and xylA were not detected in any of the genomes evaluated. In fact, this study did not associate a gene or group of genes with documented probiotic functions for the *L. plantarum* strains. For instance, the list of strains that harbored any of these 12 genes did not include the model probiotic *L. plantarum* WCFS1 [40]. Actually, *L. plantarum* carries four *bsh* genes (*bsh* 1, *bsh* 2, *bsh* 3, and *bsh* 4), which could function in place of *bshA* [42], and the *opuA* operon from *Bacillus subtilis* is homologous to the *gbu* operon [43]. In this study, the genomes harbored genes coding for proteins involved in withstanding environmental stresses. This also supports our previous findings that the strains possess the ability to tolerate acidic conditions (e.g., at pH 3) and high bile salt concentration (0.5%) [19].

The CAZy data set anticipated five significant classes of sugars in the genomes of the strains under study, i.e., GTs, GHs, CEs, CBMs, and AAs. The existence of these CAZymes helps the strains in survival, competitiveness, and persistence within the host. Because these genes are involved in the metabolism and assimilation of complex non-digestible carbohydrates, they are crucial for the bacteria’s adaptation to the gastrointestinal environment and its interaction with the host [44]. GTs are crucial for the catalysis of the transfer of sugars from activated donor molecules to acceptors and are very important for the formation of surface structures, which are recognized by host immune systems [45]. GTs can also produce structures similar to mucins by making O-linked glycosylations on serine residues [46]. CBMs can enhance the catalytic activity of the CAZymes on the substrate by binding to the substrate of the CAZymes [46]. Hence, we assume that the existence of these CAZymes helps the strains in their survival, competitiveness, and persistence within the host. In our previous study, we have shown that these strains have strong immunostimulatory activity in the human THP1-Dual™ reporter monocytes through activation of nuclear factor kappa B (NF-κB) and interferon regulatory factor (IRF) pathways [19]. This could possibly be attributed to the production of EPS associated with the T cell receptors signaling pathway by the enzyme-coding genes harbored in the genome of the strains under study.

Four fundamental regions in the genome of *L. plantarum* 55A were identified to produce bacteriocins and secondary metabolites including cyclic-lactone autoinducer (postulated to have an effect in quorum sensing to assess their cell density to regulate the production of adhesins used for biofilm formation as well as enzymes involved in the utilization of different sugars) [47], RiPP-like molecules (exhibit antibacterial activity) [48], T3PKS (produce secondary metabolites with diverse biological activities, including antimicrobials) [49], and terpenes (have antimicrobial, antiparasitic, antiallergenic, antispasmodic, antihyperglycemic, anti-inflammatory, and immunomodulatory properties) [50]. The identification of these four categories of compounds led to the notion that *L. plantarum* 55A does indeed have a potential for being used as a probiotic [51], although the exact beneficial role of these predicted properties remains to be ascertained in follow-up mechanistic studies. Our findings are in agreement with previous studies [47] that hinted cyclic peptides, similarly to the cyclic lactone autoinducer peptide, govern critical pathways of signal transduction, further targeting the polysaccharide biosynthesis and sugar utilization enzymes. Another study [51] also reported the same four regions from the *L. plantarum* 13-3 genome that were identified to produce four bacteriocins and secondary metabolites as those identified in the *L. plantarum* 55A genome. Similarly, the strains *L. plantarum* 54B and 54C had three identical regions in their genomes to produce bacteriocins and secondary metabolites (T3PKS, terpene, and cyclic-lactone autoinducer). The bacteriocin gene-detection software BAGEL v5 also showed the presence of two areas of interest for sactipeptides and plantaricins with antibacterial activities in the genome of *L. plantarum* 55A, indicating promising capability of the strain in future application as probiotics. The presence of six bacteriocin-like structures encoding genes (*PlnA*, *PlnE*, *PlnF*, *PlnJ*, *PlnK*, and *PlnN*) in the genome of *L. plantarum* 55A makes the strain similar to *L. plantarum* C11 [52] in that respect. *PlnA* is a peptide pheromone that induces the production of two peptide bacteriocins, *PlnEF* and *PlnJK*. It also has a membrane-permeabilizing, strain-specific antimicrobial effect [53,54]. *PlnEF* and *PlnJK* are class IIb two-peptide bacteriocins that require about equimolar amounts of the monomers in order to obtain maximal antimicrobial activity [55]. Meanwhile, no function could be determined for the protein encoded by *PlnN* [56].

A generally applicable operational definition of strain with a strong biological basis has not yet been provided and may not exist [57]. In theory, genomes with as little as one SNV difference may be considered to be different strains. Nonetheless, because of the overwhelming amount of strains that would result from metagenomic data, this method is not usually recommended [57]. There are no standards governing how many SNVs constitute a different strain or whether such SNVs must be fixed in the population or affect phenotypic [57]. Some authors set a cut-off of less than or equal to two SNV differences [58], while others set an ANI value of greater than 98% [59] for isolates to be considered to come from the same natural strain. The genomes of our two closely related strains, *L. plantarum* 54B and 54C (isolated from the same fermentation and the same plate), had the same (number, family) CAZymes profiles, three identical BGCs, and an ANI value of 99.9941%. However, the genomic analyses revealed differences in the location of BGCs and high SNV differences (111), indicating that these isolates are closely related but different strains. This finding also meets the regulatory requirement set in the EFSA Guidance document [32] and the EFSA’s statement [16] for an unequivocal taxonomic identification at the strain level.

LAB can also enhance the nutritional content of fermented foods by producing vitamins and cofactors, which contribute to functional food. Riboflavin, also called vitamin B2, is a water-soluble vitamin that serves as the precursor of the two essential coenzymes flavin adenine dinucleotide (FAD) and flavin mononucleotide (FMN), which are essential in the redox reactions within the cell. The riboflavin biosynthetic pathway contains seven distinct enzymes, namely, GTP cyclohydrolase II, 3,4-dihydroxy-2-butanone 4-phosphate synthase, pyrimidine deaminase/reductase, phosphatase, lumazine synthase, and riboflavin synthase; these catalyze the reaction, starting with one guanosine triphosphate (GTP) and two molecules of ribulose 5-phosphate (Ru5P) as the initial precursors [60]. Humans are not able to synthesize vitamin B2, so it must be obtained from diet [61]. Riboflavin can be produced by many microorganisms, including fungi (such as yeast) and bacteria. Indeed, the complete genome sequence of *L. plantarum* SK151 isolated from kimchi is reported to harbor a complete *rib* operon [62], and the *Limosilactobacillus fermentum* KUB-D18 genome is also reported to contain genes majorly involved in the metabolism of cofactors and vitamins including riboflavin [63]. Vitamin production by LAB varies considerably, being a species-specific or strain-dependent trait. Hence, the genetic information for riboflavin biosynthesis is species and/or strain-specific traits in LAB [64]. As a result, the inability to produce riboflavin by LAB is not an uncommon phenomenon. For example, the sequenced genome of the model probiotic *L. plantarum* strain WCFS1 contains an incomplete *rib* operon [65]. The genomes analyzed in the present work, however, harbored all genes required for riboflavin production, suggesting the isolates’ potential as probiotics. Genome analysis with the KEGG database also showed that the strains *L*. *plantarum* 54C and 55A have all enzymes of the Folate biosynthesis pathway, indicating the strains’ ability in the application of food fortification.

Finally, one of the most important findings of this study is the lack of a resistome and virulome in the strains studied, and this is consistent with another study that reported the non-pathogenicity of the *L. plantarum* strain [51]. It is very important to verify that LAB strains to be consumed as a probiotic lack virulence factors and acquired antimicrobial resistance properties prior to considering them safe for human and animal consumption [66]. The natural resistance of probiotic strains may be advantageous as it promotes both therapeutic and preventive benefits when concomitantly administered with antibiotics and facilitates intestinal microbiota recovery [67]. Overall, the lack of a resistome and virulome, in addition to the previously confirmed in vitro functional capabilities of the strains, [19] opens an avenue for a wide spectrum of research with regard to human health-related applications of the bacteria.

## 5. Conclusions

This study reported the genome sequences of three *L. plantarum* strains isolated from Ethiopian traditional cottage cheese, a rich source of the LAB strains. The results obtained in this study, with the previous in vitro research conducted, demonstrate the potential of cheese-origin *L. plantarum* strains as candidate probiotics. The in silico genomic analysis of the strains revealed the presence of putative gene clusters coding for stress resistance, adhesion, cyclic lactone autoinducer, terpenes, T3PKS, and RiPP-like gene clusters, as well as sactipeptides and plantaricins, evidencing their role as probiotics. The genomes under study also harbor all genes required for riboflavin production. Moreover, none of the strains evaluated proved to have antibiotic resistance genes or virulence factors, suggesting their potential safety for probiotic applications. Collectively, the genomic information guarantees the safe use of these strains as probiotics and opens new possibilities to exploit the health-promoting potential of the strains.

## Figures and Tables

**Table 1:** Comparison of chromosomal properties of *L. plantarum* isolates.

Isolate	54B	54C	55A
Completeness (%)	99.07	99.07	99.07
Genome size (bp)	3,398,069	3,372,323	3,299,167
N50 (contigs)	105,937	131,701	169,607
Contigs	156	148	77
GC content (%)	44.3	44.3	44.5
Number of ambiguous bases	100	100	0
Coding sequence (CDS)	3259	3230	3108
Genes	3335	3306	3185
tmRNA	1	1	1
rRNA	7	7	7
tRNA	68	68	69
Species	*L. plantarum*	*L. plantarum*	*L. plantarum*

**Table 2:** Average nucleotide values of the strains.

Strains Compared	ANI Values (%)
54B vs. 54C	99.9941
54B vs. WCFS1	98.7873
54B vs. 55A	98.6644
54C vs. WCFS1	98.7732
54C vs. 55A	98.7297
55A vs. WCFS1	98.8804

**Table 3:** Characteristics of biosynthetic gene clusters of the isolates *L. Plantarum* 54B and 54C.

Secondary Metabolite 1: PKS					
isolate	region	BGC	Core gene	Location (nt)	Total nucleotide (nt)
54B	2.1	PKS	T3PKS	1–34,310 nt	34,310 nt
54C	2.1	PKS	T3PKS	175,611–209,976 nt	34,366 nt
Secondary metabolite 2: terpene					
54B	4.1	terpene	terpene	140,604–161,485 nt	20,882 nt
54C	4.1	terpene	terpene	26,228–47,109 nt	20,882 nt
Secondary metabolite 3: RiPP					
54B	40.1	RiPP	Cyclic-lactone-autoinducer	1239–17,049 nt	15,811 nt
54C	41.1	RiPP	Cyclic-lactone-autoinducer	1–15,811 nt.	15,811 nt

**Table 4:** Riboflavin biosynthesis pathway genes present in the *L. plantarum* 54B (A) and 55A (B) genomes.

**(A) Genes Harbored According to the KEGG Database in *L*. *plantarum* 54B**	**Gene Ontology (GO) in Bv-Brc.Org**	**Contig**	**Start**	**End**	**Length, Bp**
FMN adenylyltransferase (EC 2.7.7.2)/Riboflavin kinase (EC 2.7.1.26)	GO:0003919/GO:0008531	1590.4076.con.0001	12,980	13,828	849
Diaminohydroxyphosphoribosylaminopyrimidine deaminase (EC 3.5.4.26)/5-amino-6-(5-phosphoribosylamino)uracil reductase (EC 1.1.1.193)	GO:0008835/GO:0008703	1590.4076.con.0002	39,165	40,232	1068
Riboflavin synthase eubacterial/eukaryotic (EC 2.5.1.9)	GO:0004746	1590.4076.con.0002	40,233	40,835	603
3,4-dihydroxy-2-butanone 4-phosphate synthase (EC 4.1.99.12)/GTP cyclohydrolase II (EC 3.5.4.25)	GO:0008686/GO:0003935	1590.4076.con.0002	40,837	42,051	1215
6,7-dimethyl-8-ribityllumazine synthase (EC 2.5.1.78)		1590.4076.con.0002	42,048	42,527	480
FMN adenylyltransferase (EC 2.7.7.2)/Riboflavin kinase (EC 2.7.1.26)	GO:0003919/GO:0008531	1590.4076.con.0003	47,566	48,567	1002
Alkaline phosphodiesterase I (EC 3.1.4.1)/Nucleotide pyrophosphatase (EC 3.6.1.9)	GO:0004528/GO:0004551	1590.4076.con.0009	34,650	35,945	1296
**(B) Genes Harbored According to the Kegg Database in *L. Plantarum* 55A**	**Gene Ontology (GO) in Bv-Brc.Org**	**Contig**	**Start**	**End**	**Length, Bp**
FMN adenylyltransferase (EC 2.7.7.2)/Riboflavin kinase (EC 2.7.1.26)	GO:0003919/GO:0008531	1590.4078.con.0007	37,971	38,819	849
FMN adenylyltransferase (EC 2.7.7.2)/Riboflavin kinase (EC 2.7.1.26)	GO:0003919/GO:0008531	1590.4078.con.0018	14,036	15,037	1002
Diaminohydroxyphosphoribosylaminopyrimidine deaminase (EC 3.5.4.26)/5-amino-6-(5-phosphoribosylamino)uracil reductase (EC 1.1.1.193)	GO:0008835/GO:0008703	1590.4078.con.0022	37,364	38,431	1068
Riboflavin synthase eubacterial/eukaryotic (EC 2.5.1.9)	GO:0004746	1590.4078.con.0022	38,432	39,034	603
3,4-dihydroxy-2-butanone 4-phosphate synthase (EC 4.1.99.12)/GTP cyclohydrolase II (EC 3.5.4.25)	GO:0008686/GO:0003935	1590.4078.con.0022	39,036	40,250	1215
6,7-dimethyl-8-ribityllumazine synthase (EC 2.5.1.78)		1590.4078.con.0022	40,247	40,726	480
Alkaline phosphodiesterase I (EC 3.1.4.1)/Nucleotide pyrophosphatase (EC 3.6.1.9)	GO:0004528/GO:0004551/GO:0004551	1590.4078.con.0023	24,577	25,872	1296
3,4-dihydroxy-2-butanone 4-phosphate synthase (EC 4.1.99.12)/GTP cyclohydrolase II (EC 3.5.4.25)	GO:0008686/GO:0003935	1590.4078.con.0029	11,149	11,688	540

## Data Availability

All data generated from this study, except the sequence data, are either included in the manuscript or provided as Supplementary Materials. The sequence data are deposited in the NCBI database.

## Supplementary Materials

The following supporting information can be downloaded at https://www.mdpi.com/article/10.3390/genes15111389/s1, Table S1: Characteristics of the 23 *L. plantarum* used in this study [36,68,69,70,71,72,73,74,75,76,77,78,79,80,81,82,83,84,85,86,87,88,89], Table S2: Probiotic marker genes (PMGs) in *L. plantarum* shell and cloud genome [40], Table S3: Probiotic marker genes.
