# Supplementary material for: Genomic Characterization of Lactiplantibacillus plantarum Strains: Potential Probiotics from Ethiopian Traditional Fermented Cottage Cheese
Source: Genes (Basel). 2024 Oct 29;15(11):1389. doi: 10.3390/genes15111389 (PMC11593849; doi:10.3390/genes15111389)
Supplement: Supplementary file 1 [file genes-15-01389-s001.zip › genes-3233537-supplementary.pdf]

Supplementary Table S1: Characteristics of the 23 *L. plantarum* used in this study

Strain name	Genbank accession	Ecological niche	Genome Status	N. of contigs	Citation
WCFS1	AL935263.2	Human saliva	Complete	1	[68]
ZJ316	CP004082	Infant faeces	Complete	1	[69]
WLPL04	LKCO00000000	Human isolate	Complete	1	[70]
HFC8	CP012650	Human isolate	Complete	11	[71]
LZ95	CP012122	Infant faeces	Complete	3	[72]
CMPG5300	AXZV01000000	Vaginal isolated	Draft	48	[73]
P8	CP005942	Dairy isolate	Complete	1	[74]
LZ206	CP015966	Dairy isolate	Complete	4	[75]
LZ227	CP015857	Dairy isolate	Complete	6	[76]
K25	CP020093	Dairy isolate	Complete	7	[77]
YW11	CP035031	Dairy isolate	Complete	6	[78]
RI-113	CP017406	Meat Product	Complete	7	[79]
TMW 1.25	CP017354	Meat Product	Complete	7	[80]
MF1298	CP013149	Meat product	Complete	15	[81]
B21	CP010528	Meat product	Complete	29	[82]
LPL-1	CP021997	Fish isolate	Complete	2	[83]
LP3	CP017066	Vegetables	Complete	3	[36]
ZS2058	CP012343	Sauerkraut	Complete	1	[84]
TMW 1.1478	CP021932	Honey	Complete	2	[85]
ST-III	CP002222	Kimchi	Complete	1	[86]
JDM1	CP001617	Grass silage	Complete	1	[87]
16	NCIMB41875	Malt production steep water	Complete	11	[88]
JDARSH	PYBS00000000	Sheep Milk	draft	31	[89]
